# Epigenetic reaction norms: possible but not inevitable

**DOI:** 10.1093/emph/eox015

**Published:** 2018-02-05

**Authors:** Stephen Stearns

**Affiliations:** Department of Ecology and Evolutionary Biology, Yale University, Box 208106, New Haven, CT 06520, USA

I found the analyses and recommendations in Lea *et al.* (2017) mostly interesting, competent and sound but wish that more guidance had been given for future research. Which empirical results would advance knowledge most at this point, and why those priorities rather than others? That question is not clearly answered. Neither did the authors ask, what empirical puzzles could be solved by the research they suggest, and if those puzzles were solved, what difference would that make? Is there an empirical crisis calling urgently for a solution, or are they proposing more modest steps? The authors provide partial answers to these important questions but do not pose them sharply enough to motivate as many researchers as they might have. Taking a clear stand, even if it proves wrong, is often helpful. I wish they had been more provocative.

The authors could have made greater use of the concept of a reaction norm [1], which is more familiar to evolutionary biologists than to clinicians. They used reaction norms explicitly in their Fig. 2, but only mentioned the concept in the caption to Fig. 1. If they had engaged with it, they might have hit upon the idea of defining epigenetic reaction norms as the mapping of a given epigenome onto a set of phenotypes as a function of the environment. That would have given them the handle needed to distinguish between maternal epigenetic reaction norms induced by environmental conditions experienced by the mother but expressed in the offspring and the epigenetic reaction norms induced directly in the offspring by environmental conditions experienced by the offspring early in life. With that important distinction in mind, they could then have suggested straightforward experiments in model organisms to distinguish how much of the response of the offspring was induced by information received by the mother in the previous generation and how much was induced later by information received directly by the offspring. Such experiments would give us important insights into the timescales of the plastic responses and how they match, or mismatch, environmental variation. They should initially be done in clonal model organisms so that DNA sequence variation can be nearly fixed, and responses can be confidently treated as epigenetic. There is a small literature on maternal effect reaction norms [2]; it can be used to guide new experiments that can now exploit the tools of epigenomics.

The concept of epigenetic reaction norms can be extended to encompass a series of conditions encountered in the life of a single individual. What impact does variation in environmental conditions in the first 5% of normal lifespan have on plastic responses to environmental conditions at 10%, 20%, … of lifespan?

Let me recall two empirical results that are apropos. The first comes from experiments on mosquito fish from Galveston, TX, USA [3, 4]. The environment experienced in the first week of life (fresh water vs. brackish water) induced the production of broods of offspring over 100 days later that matched the size and number of offspring found in that environment in nature. If the differences in size and number of offspring were adaptively matched to differences between the freshwater and brackish water environments, then those mosquito fish had evolved a predictive adaptive response. Their populations are large, genes are continually moving across the fresh-brackish boundary, and these populations have plausibly been exposed to that contrast for thousands of generations. Such are the conditions under which a predictive adaptive response might evolve.

The second result comes from experimental evolution on fruit flies and has not previously been reported. In our 7-year experiment that examined the effects of high versus low adult mortality on lifespan and aging [5], there was a second, previously unreported set of treatments. They associated a chemical cue experienced by the larvae with an adult mortality regime. Cues and mortality regimes alternated in time. In one treatment, the cue indicated that high adult mortality could be expected. In the other, the same cue indicated that low adult mortality could be expected. In neither treatment did the flies evolve a plastic response to the cue, which we knew they could perceive and was reliably associated with a very strong difference in adult mortality (expected adult lifespan of 1 day vs. 6 weeks). In the same period of time, about 100 generations, the flies in the published treatments [6] did evolve the expected differences in lifespan, age and size at eclosion and fecundity early in life. Either there was not enough genetic variation for plasticity in response to the chemical cue that we chose, or the populations were too small for the signal of selection to overcome genetic drift, or the physiological and developmental systems of the flies were otherwise constrained and could not evolve the appropriate response. The lesson of that experiment is that predictive adaptive responses may not evolve rapidly for any of a variety of reasons.

## Funding

This work was funded by Yale University.
